# A Comparative Analysis for Inhospital and Postdischarge Puerperal Sepsis (Readmissions) Among Mothers Admitted in Mbarara Regional Referral Hospital

**DOI:** 10.7759/cureus.109040

**Published:** 2026-05-17

**Authors:** Rachel Luwaga, Deborah Shindell, Francis Bajunirwe, Joseph Ngonzi

**Affiliations:** 1 Department of Nursing, Mbarara University of Science and Technology, Mbarara, UGA; 2 Department of Nursing, University of Nevada, Reno School of Medicine, Reno, USA; 3 Department of Community Health, Faculty of Medicine, Mbarara University of Science and Technology, Mbarara, UGA; 4 Department of Obstetrics and Gynecology, Mbarara University of Science and Technology, Mbarara, UGA

**Keywords:** cesarean section, inhospital sepsis, maternal sepsis, postpartum infection, puerperal sepsis, puerperal sepsis after discharge, readmission

## Abstract

Introduction: Puerperal sepsis is defined as an infection of the genital tract occurring within 42 days after delivery. It is characterized by symptoms such as chills, fever, foul-smelling vaginal discharge, pelvic pain, and delayed uterine involution. The condition is more prevalent in low-income countries, with Uganda among those reporting higher rates. Puerperal sepsis can be acquired either during the initial birth hospital admission or after discharge, resulting in readmission. Although the risk of infection exists during the antenatal period, it increases significantly during delivery and remains elevated throughout the postnatal period.

Knowledge of self-care and use of postnatal care services are often limited in low-resource settings, increasing the risk of infection. Understanding the differences between mothers readmitted and those who acquire puerperal sepsis while hospitalized provides information for designing targeted interventions in its prevention. Therefore, we compared the magnitude, maternal characteristics, and associated factors among mothers who developed puerperal sepsis during their initial admission and those readmitted with postdischarge puerperal sepsis at Mbarara Regional Referral Hospital.

Methods: A retrospective chart review was conducted on a total of 316 files from 2017 to 2024 of mothers diagnosed with puerperal sepsis who had been admitted to the maternity and gynecology wards of Mbarara Regional Referral Hospital. Information was extracted using a predesigned checklist. The data were then cleaned, entered into MS Excel (Microsoft Corporation, Redmond, Washington, United States), and imported into IBM SPSS Statistics for Windows, Version 20 (Released 2011; IBM Corp., Armonk, New York, United States) for analysis. Readmissions were summarized as proportions, and binary logistic regression was performed for both bivariate and multivariate analyses. Additionally, the Mann-Whitney U test was used to assess differences in length of hospital stay.

Results: The proportion of readmissions (66.8%, n = 211) was higher than that of mothers who acquired puerperal sepsis while still hospitalized. Most readmitted mothers had delivered by cesarean section (86.5%, n = 186) and were from rural areas (67.3%, n = 142). Factors significantly associated with readmission included age 15-19 years (adjusted odds ratio (AOR): 6.815; 95% CI: 1.786-26.018) and being primiparous (AOR: 0.424; 95% CI: 0.186-0.968). There was also a significant difference in length of hospital stay between readmitted and nonreadmitted mothers (Mann-Whitney U: 7974; Z: −4.085; p < 0.001).

Conclusion and recommendation: Despite the observed reduction in admissions due to puerperal sepsis, the predominance of postdischarge cases, particularly among mothers who underwent cesarean section and younger mothers, remains a significant challenge to achieving Sustainable Development Goal 3. Targeted interventions focusing on the prevention of puerperal sepsis, with emphasis on postdischarge self-care, are therefore essential.

## Introduction

Puerperal sepsis remains one of the leading causes of maternal mortality worldwide, and many of the deaths associated with it occur at home, making them difficult to detect and often underreported [1]. It is defined as an infection of the genital tract occurring within 42 days after delivery. Clinically, it presents with symptoms such as chills, fever, foul-smelling vaginal discharge, pelvic pain, and delayed uterine involution [2]. Although largely preventable, puerperal sepsis is still reported as the second leading cause of maternal mortality, accounting for 19.2% of all maternal deaths worldwide.

Globally, the prevalence of puerperal sepsis is estimated at 15% [3], with disproportionately higher rates in low-income countries such as Uganda, where it reaches approximately 400 cases per 100,000 live births, compared to 9-49 per 100,000 deliveries in high-income countries [4]. This translates to about one in 8,000 women developing puerperal sepsis in high-income settings versus approximately one in 66 mothers in low-income settings [5]. In Uganda, maternal sepsis accounts for 13% of all maternal deaths, second to hemorrhage [6].

Encouragingly, Uganda has recorded a significant decline in maternal mortality due to sepsis, from 16.5% in 2020 to 8.6% in 2022 [6]. This progress is largely attributed to interventions implemented by the Ministry of Health, including the introduction of the Maternal and Perinatal Death Surveillance and Response (MPDSR) system, which supports surveillance, notification, auditing, and, importantly, learning from the causes of maternal deaths to prevent future occurrences. In addition, the government has strengthened the implementation of Water, Sanitation, and Hygiene (WASH) guidelines to improve hygiene practices in health facilities and reduce the risk of infection during delivery [6].

Despite the implementation of these interventions, cases of puerperal sepsis continue to occur both within health facilities during initial birth hospital admission and after discharge (readmission). Readmissions related to these infections are often underreported, resulting in limited data on their magnitude and the factors associated with them.

Hospital readmissions have been associated with multiple points along the continuum of maternal care. Although the risk of infection begins during the antenatal period, it rises significantly during delivery and remains elevated throughout the postnatal period (up to 42 days after birth), largely influenced by the mode of delivery and the quality of care provided [7]. Inadequate infection prevention practices during these stages, both in health facilities and at home, increase the likelihood that mothers will develop infections that may ultimately result in hospital readmission [8].

The cumulative risk of postdelivery readmission has been categorized into two periods: days 0-7 and days 8-28, corresponding to early (0-7 days) and late (8-28 days) readmissions [9]. Alternatively, some definitions describe early readmission as admission with a diagnosis of sepsis within six weeks postpartum, and late readmission as occurring beyond six weeks up to nine months postpartum [10].

Sepsis has been associated with a fourfold increase in both early and late postnatal hospitalizations. In California, 52% of mothers experienced early readmissions, while 44% had late readmissions [11]. Additionally, studies in the United States have reported an increase in readmissions due to puerperal sepsis from 9% to 11% between 2007 and 2018 [12]. Given these high rates in a high-income setting, it is likely that the burden is even greater in low-income countries with limited resources, highlighting potential gaps in postnatal care.

There is limited evidence distinguishing between mothers who develop puerperal sepsis during hospitalization and those who acquire it after discharge. A clearer understanding of the magnitude, maternal characteristics, and associated factors in each group is essential for identifying critical gaps in care and informing targeted, comprehensive interventions to prevent puerperal sepsis both within health facilities and after discharge. This study, therefore, aimed to compare the magnitude, maternal characteristics, and associated factors of inhospital and postdischarge puerperal sepsis (readmissions) among mothers admitted to Mbarara Regional Referral Hospital.

## Materials and methods

Study design

This study employed a retrospective chart review design.

Study setting

Mbarara Regional Referral Hospital (MRRH) has approximately 350-bed capacity and serves as a teaching hospital for a number of medical and nursing schools in the Mbarara region of Uganda. It is the largest referral hospital in Southwestern Uganda, serving a population of 4.6 million people living in predominantly rural, agrarian settings with a catchment area of more than 11 districts. MRRH provides general and specialized care and has the capacity to carry out diagnostic and therapeutic investigations.

Population

Mothers admitted to the MRRH with puerperal sepsis from 2017 to 2024.

Inclusion Criteria

**Figure 1:**
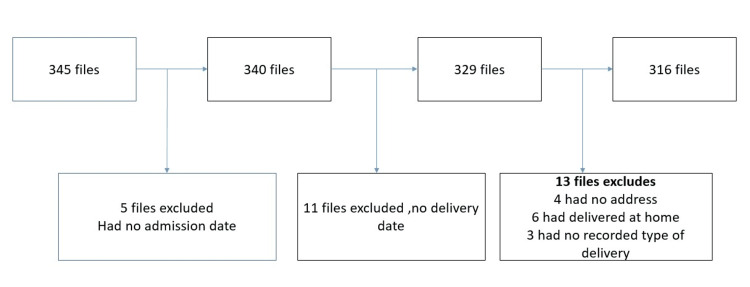
File inclusion and exclusion process

All charts for mothers with 90% of the data, which included demographic characteristics, age, address, parity, date of delivery, admission date for puerperal sepsis admission, and discharge date in MRRH, were included in the study (Figure 1). 

Exclusion Criteria

Charts for mothers missing more than 10% of data required, including demographic characteristics, age, address, date of delivery, type of delivery, date of admission, and discharge, were excluded from the study (Figure 1).

Sample size

The sample size was calculated using the Kish and Leslie formula (1965) model, and an anticipated prevalence of puerperal sepsis of 39% [13] with 365 files; however, only 316 files met the inclusion criteria.

Sampling method

This study used a census approach, including all files that met the inclusion criteria for data retrieval and analysis.

Data collection tool

A 35-item data retrieval checklist created from literature from a study on readmissions due to maternal sepsis [9] was used to extract information from the patient files. The items on the checklist included the mother’s age, address, parity, date of admission for puerperal sepsis, date of delivery, place of delivery, mode of delivery, HIV status, baby’s status, referral status, and outcomes following admission.

Content validity was established through review by one obstetrician and two senior obstetric residents, and a validity coefficient was obtained. All questions were found relevant to the study, and the content validity statistic was 0.91. Reliability testing was conducted using data from a pretest of 10 files from the 2025 admission records, yielding a Cronbach’s alpha coefficient of 0.79.

All files lacked information on level of education, occupation, marital status, and comorbidities; therefore, these variables were removed from the study tool.

Data collection procedure

Using the maternity and gynecology ward registers, inpatient (IP) numbers for all mothers diagnosed with puerperal sepsis between 2017 and 2024 were identified and recorded together with their names and ages. A record sheet was then used to manually retrieve the corresponding files from the records office based on each IP number and year.

Puerperal sepsis diagnosis was confirmed from the documentation by clinicians as infection of the genital tract from birth to 42 days after birth, with signs of high fever (>38°C), lower abdominal pain, and foul-smelling vaginal discharge (lochia). Data from the files was obtained by reading the files and was recorded in the checklists.

Data management and analysis

The data was collected and entered into MS Excel (Microsoft Corporation, Redmond, Washington, United States), where they were cleaned and organized for analysis. It was then entered into IBM SPSS Statistics for Windows, Version 20 (Released 2011; IBM Corp., Armonk, New York, United States) and analyzed. Demographic characteristics were represented in tables as percentages and frequencies. Due to the skewed nature of the data, binary logistic regression was performed to determine the likelihood of association, expressed as odds ratios with 95% confidence intervals and a p-value of 0.05. Multivariate analysis was further conducted to ascertain the predictors of readmission, from which adjusted odds ratios were obtained. 

Outcome variables such as length of stay were summarized using the median and interquartile range, and the difference in length of stay between the groups was assessed using the Mann-Whitney U test. Other outcomes, including mortality, recovery, and surgery, were described using proportions.

Ethical considerations

A waiver of consent was obtained from the Mbarara University of Science and Technology Research Ethics Committee (MUST-2024-1696) to access and collect data retrospectively from patient files. Permission to conduct the study was also obtained from the Uganda National Council for Science and Technology (HS5504ES). Permission was obtained from Mbarara Regional Referral Hospital to access the records of the hospital.

## Results

A total of 316 charts met the inclusion criteria.

Demographic characteristics

**Figure 2:**
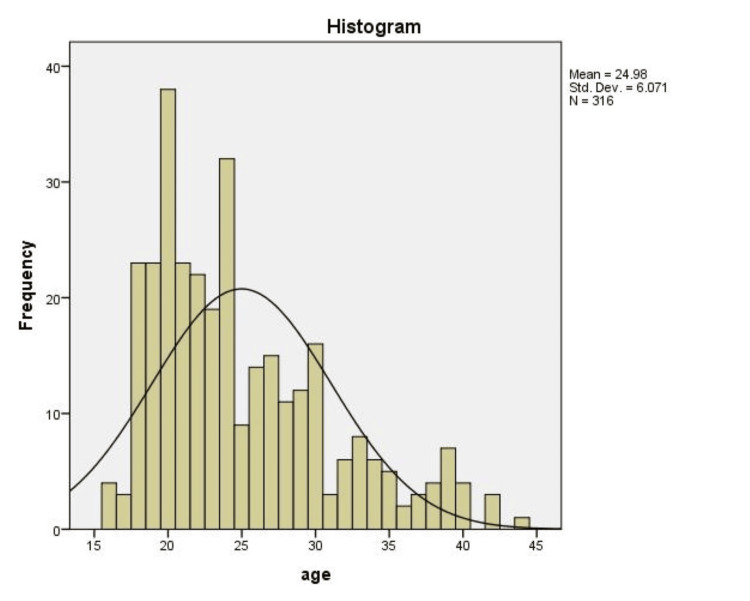
Histogram showing distribution of age

The population was positively skewed (skewness: 0.952; kurtosis: 0.242), with the majority of the participants below 30 years of age, with a median age of 24, a mode of 20, a range of 16 to 44 years, and an interquartile range of 9 (Figure 2).

The majority of mothers (235, 74.4%) were aged 20-34 years. Adolescents aged 15-19 years also contributed a notable proportion of mothers with puerperal sepsis (53, 16.8%). Most participants were from rural settings (217, 68.7%), were primiparous (164, 51.9%), and had delivered by cesarean section (283, 89.6%). Additionally, the majority had a negative HIV status (285, 90.2%), were delivered in a hospital (222, 70.3%), and had live births (244, 77.2%) (Table 1).

**Table 1:** Demographic characteristics of the mothers Hospital includes private and government hospitals

Characteristics	Frequency	Percentage
Age category		
15-19 years	53	16.8
20-34 years	235	74.4
≥ 35 years	28	8.9
Address		
Rural	217	68.7
Urban	99	31.1
Parity		
Primipara	164	51.9
Multipara	104	32.9
Grand multipara	48	15.2
Type of delivery		
Spontaneous vaginal delivery	33	10.4
Cesarean section	283	89.6
Birth facility		
Hospital	222	70.3
Health center	80	25.3
Private clinic	14	4.4
Sero status		
TR	285	90.2
TRR	20	6.3
Unknown	11	3.5
Status of the child		
Alive	244	77.2
Dead	68	21.5
Unknown	4	1.3
Outcome		
Recovered	249	78.8
Runaway	50	15.8
Recovered with hysterectomy	12	3.8
Died	5	1.6

Trend in puerperal sepsis admissions over eight years

**Figure 3:**
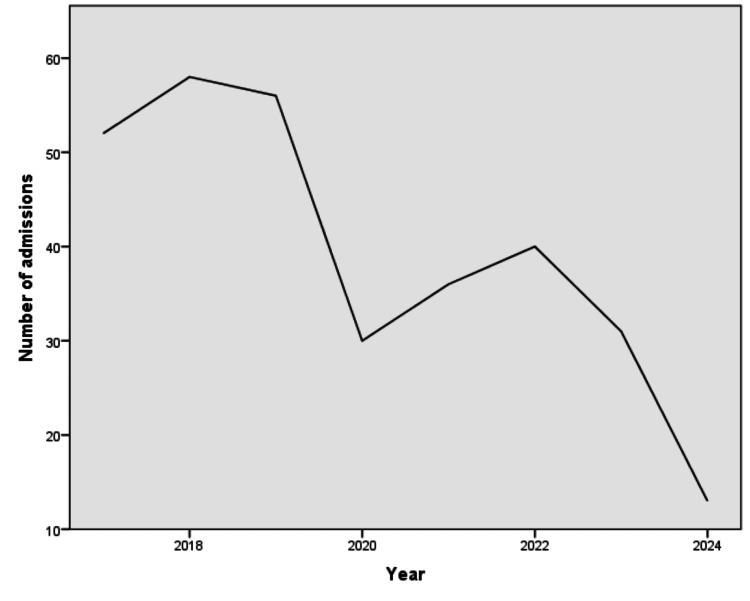
Trends of puerperal sepsis admissions over eight years

Rates of sepsis admissions were stable from 2017 to 2019 at roughly 17-18% of deliveries per year. In the years 2020-2023, the rate dropped to roughly 11-12%, then dropped again in 2024 to only 4%. There has been a significant reduction in puerperal sepsis admissions over the years, with the highest admission registered in 2018, i.e., 60 (18.6%), and the least in 2024, 13 (4%) (Figure 3).

Puerperal sepsis admission rate over eight years

**Figure 4:**
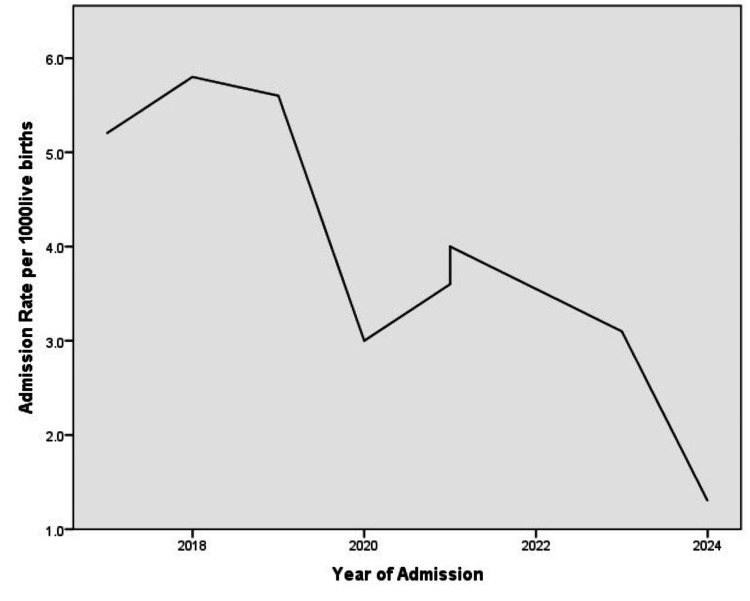
Admission rate for puerperal sepsis per 1000 live births Mbarara Regional Referral Hospital (MRRH) registers an average of 10000 live births per year.

There has been a gradual decline in the annual puerperal sepsis admission rate per 1,000 live births. The highest number of admissions was recorded in 2018 (6.0 puerperal sepsis admissions per 1,000 live births), followed by a significant reduction to 1.3 admissions per 1,000 live births in 2024 (Figure 4).

Puerperal sepsis readmission prevalence

**Figure 5:**
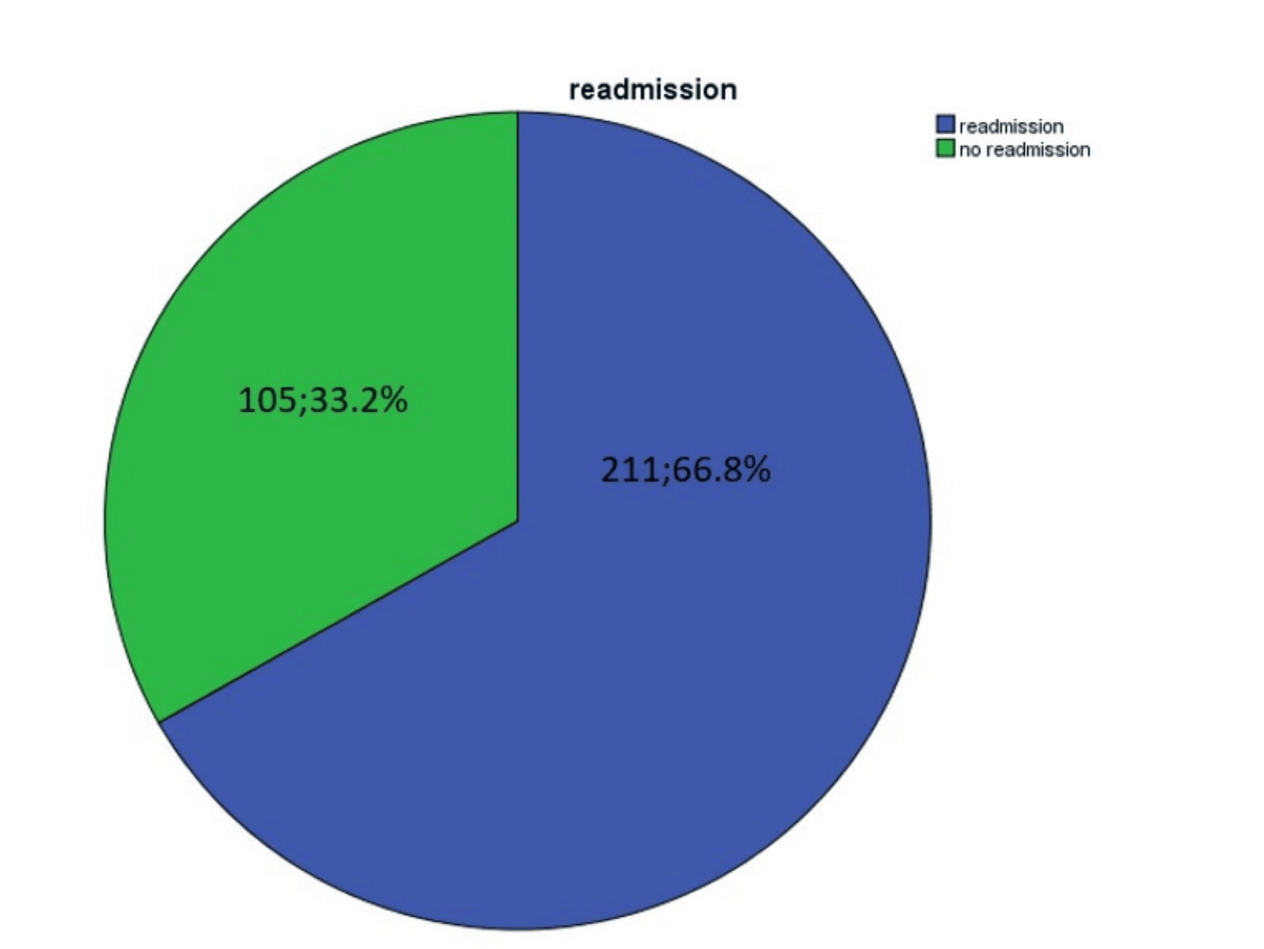
Prevalence of readmissions

The majority of mothers admitted with puerperal sepsis (211, 66.8%) acquired the infection after being discharged from the hospital following delivery (Figure 5).

Readmission prevalence over eight years

**Figure 6:**
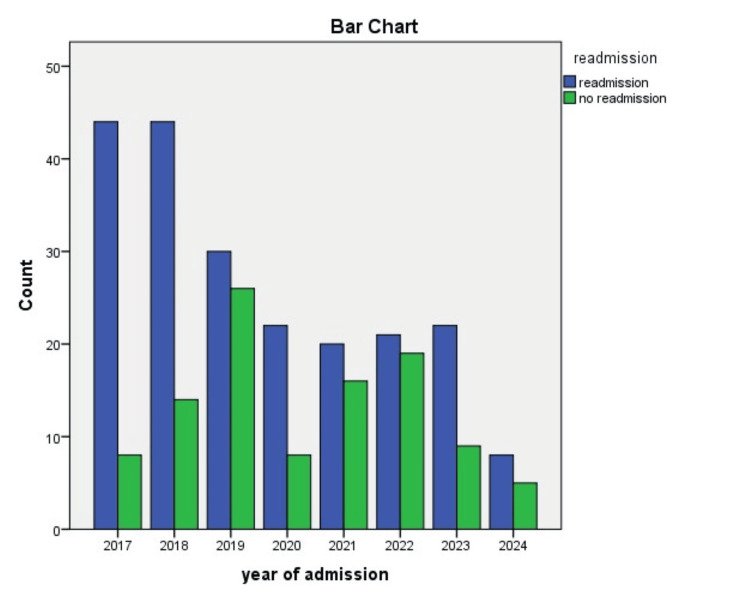
Trends in readmissions over eight years

In 2017-2018, the majority of mothers admitted for puerperal sepsis were readmissions, 44 (84.6%) and 44 (75.9%), respectively. Although there has been a reduction in overall puerperal sepsis admissions over the eight years, most mothers admitted each year were readmissions (Figure 6).

Factors associated with readmission due to puerperal sepsis

Among the readmitted mothers, 31 (14.7%) were aged 15-19 years. Mothers in this age group were six times more likely to be readmitted compared with those aged over 35 years (crude odds ratio (COR): 3.365; CI: 1.075-9.913; AOR: 6.815; CI: 1.786-26.018; p-value: 0.005)

Most readmissions (157, 74.4%) occurred among mothers aged 20-34 years, who had nearly fourfold higher odds of readmission due to puerperal sepsis compared with mothers aged 35 years and above (AOR: 4.395; CI: 1.378-14.020; p-value: 0.012)

Primiparous mothers accounted for 106 (30.7%) of the readmissions. Primiparity was statistically protective against readmission (AOR: 0.424; p: 0.042; 95% CI: 0.186-0.968). Overall, the majority of readmitted mothers (139, 65.9%) had delivered in a hospital, had cesarean section (186, 88.2%), and were residing in rural areas (142, 67.3%). There was no statistically significant association between mode of delivery and the outcome, as mothers who had spontaneous vaginal delivery (SVD) did not differ significantly from those who had cesarean section (p > 0.05) (Table 2).

**Table 2:** Factors associated with readmission due to puerperal sepsis TR: HIV negative; TRR: HIV positive; primipara: first birth; multipara:  2 to 4 births; grand multipara: 5 births and more; COR: crude odds ratio; AOR: adjusted odds ratio

	Readmission		Bivariate			Multivariate		
Independent variables	Yes n (%)	No n (%)	p-value	COR	CI	p-value	AOR	CI
Age category								
15-19 years	31 (14.7)	22 (21.0)	0.037	3.365	1.075-9.913	0.005	6.815	1.786-26.018
20-34 years	157 (74.4)	78 (74.3)	0.107	2.285	0.883-6.525	0.012	4.395	1.378-14.020
≥35 years	23 (10.9)	5 (4.8)		Ref				
Address								
Rural	142 (67.3)	75 (71.4)	0.456	1.215	0.728-2.027	0.237	1.402	0.801-2.452
Urban	69 (32.7)	30 (28.6)		Ref				
Parity								
Primipara	109 (51.7)	55 (52.4)	0.71	0.841	0.431-1.641	0.042	0.424	0.186-0.968
Multipara	72 (34.1)	32 (30.5)	0.611	0.741	0.361-1.518	0.081	0.477	0.208-1.095
Grand multipara	30 (14.2)	18 (17.1)		ref				
Type of delivery								
Spontaneous vaginal delivery	25 (11.8)	8 (7.6)	0.251	0.614	0.207-1.412	0.451	0.689	0.262-1.816
Cesarean section	186 (88.2)	97 (92.4)		ref				
Birth facility								
Health center	61(28.4)	19 (17.8)	0.805	1.142	0.288-4.524	0.919	1.076	0.259-4.479
Hospital	139 (65.9)	83 (77.6)	0.239	2.189	0.594-8.079	0.205	2.406	0.619-9.346
Private clinic	11 (5.2)	3 (2.8)	ref					
Sero status								
TR	191 (90.5)	94 (89.5)	0.394	0.591	0.176-1.985	0.201	0.423	0.311-1.581
TRR	14 (6.6)	6 (5.7)	0.392	0.514	0.112-2.361	0.249	0.388	0.075-1.937
Unknown	6 (2.8)	5 (4.8)	ref					
Status of the child								
Alive	165 (78.2)	79 (75.2)	0.466	0.479	0.066-3.462	0.489	0.486	0.063-3.760
Dead	44 (20.9)	24 (22.9)	0.557	0.545	0.072-4.120	0.593	0.565	0.070-4.589
Unknown	2 (0.9)	2 (1.9)	ref					

Descriptive statistics for hospital length of stay

**Figure 7:**
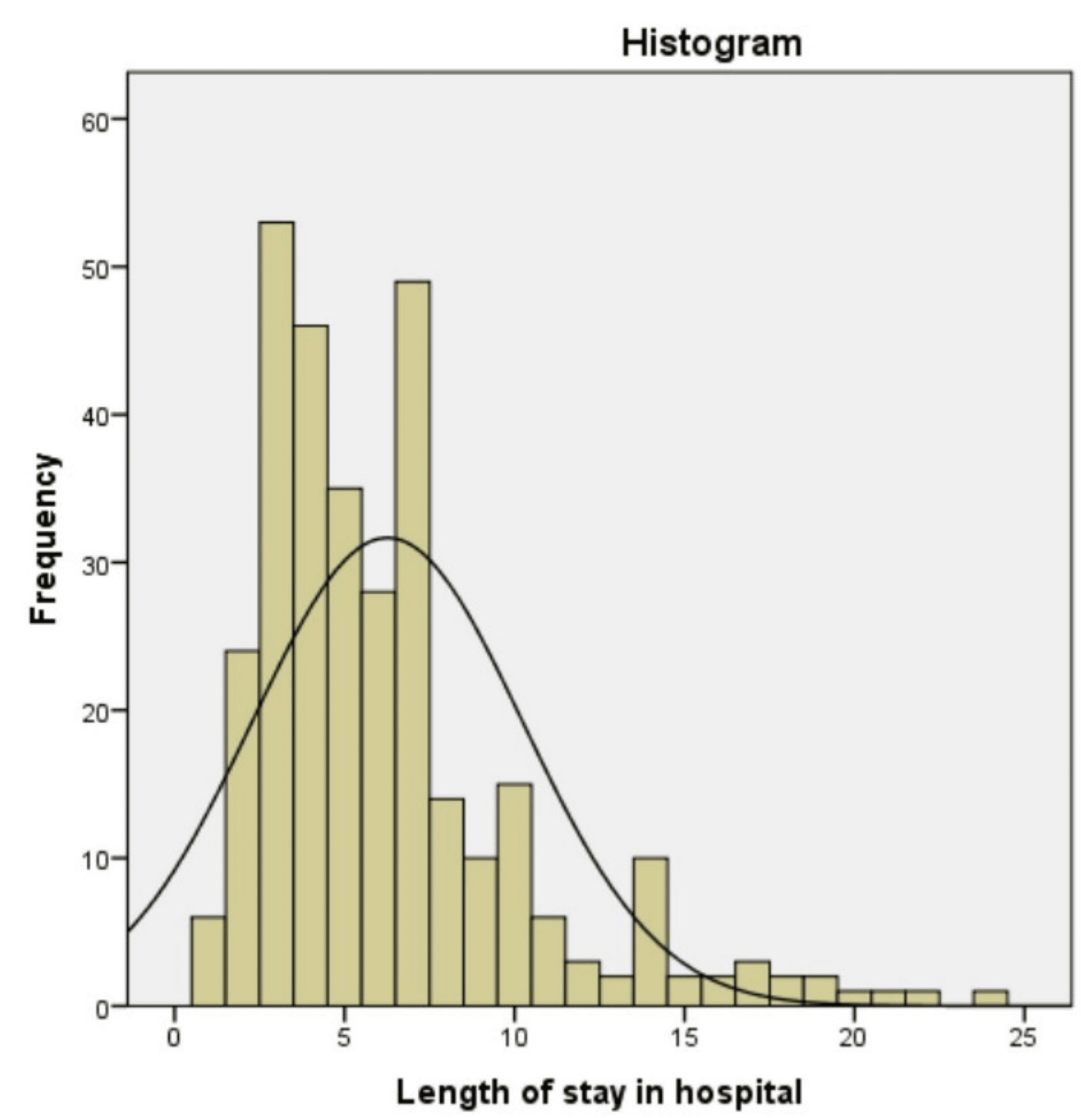
Distribution of length of hospital stay

The length of hospital stay was positively skewed, with the majority of mothers staying for less than 10 days. The median length of stay was five days, the mode was three days, with a skewness of 1.619 and a kurtosis of 3.074 (Figure 7).

Difference in Length of Stay Between the Readmission and Those Who Acquired Puerperal Sepsis in the Hospital

There was a difference in the length of hospital stay between mothers who acquired puerperal sepsis in the hospital and those who acquired puerperal sepsis after discharge and were readmitted. Mothers with postdischarge puerperal sepsis had a median length of stay of six days (interquartile range (IQR): 1-24 days), while those who acquired puerperal sepsis during hospitalization had a median length of stay of four days (IQR: 1-19 days). The Mann-Whitney U test showed a statistically significant difference in length of stay between the two groups (U = 7974; Z = −4.085; p < 0.001).

Comparison of Outcomes Between Those Readmitted and Those Who Acquired Puerperal Sepsis Inhospital

The majority of mothers in both groups recovered. However, eight (3.7%) mothers in the readmitted group underwent hysterectomy, while four (3.7%) mothers who had not been readmitted (acquired puerperal sepsis during hospitalization) died (Table 3).

**Table 3:** Comparison of the outcomes for those who were readmitted and those who acquired puerperal sepsis in the hospital

	Readmission	
Outcome	Yes n (%)	No n (%)
Recovered	168 (79.6)	81 (77.61)
Runaway/left against medical advice	34 (16.1)	16 (15.2)
Recovered with hysterectomy	8 (3.8)	4 (3.8)
Died	1 (0.5)	4 (3.8)

## Discussion

Sustainable Development Goal 3 targets a global reduction in the maternal mortality ratio to less than 70 per 100,000 live births. Low-income countries, including Uganda, have made efforts to achieve this goal by implementing interventions that address major contributors to maternal mortality, with sepsis being one of the leading causes. These efforts include updated recommendations for pregnant women to attend at least eight antenatal care visits, compared to the previous minimum of four visits recommended in 2018 [13]. These visits incorporate health education on nutrition and hygiene, although compliance remains suboptimal [6].

Additionally, the expansion of health facilities equipped to manage obstetric complications, particularly Health Center III and IV facilities, has strengthened maternal care services [6]. These combined interventions have likely contributed to the reduced burden of admissions for puerperal sepsis at Mbarara Regional Referral Hospital observed from 2017 to 2024.

The observed reduction in puerperal sepsis may also be attributed to the rollout of updated essential guidelines for the care of postcesarean section mothers, as well as the MPDSR. These guidelines emphasize strict adherence to aseptic techniques, the administration of prophylactic antibiotics for mothers undergoing emergency cesarean sections, and the use of intravenous antibiotics during the immediate postoperative period. Strengthening infection prevention and early antimicrobial therapy may have contributed significantly to the decline in sepsis-related admissions [6].

The study found that most cases of puerperal sepsis occurred after hospital discharge, leading to subsequent readmission. Similar patterns have been reported elsewhere, with comparable associated factors [12]. A higher proportion of readmitted mothers had delivered by cesarean section, often for the first time, compared to the relatively low readmission rates among those who had SVDs. This is consistent with previous studies identifying cesarean section as a major risk factor for puerperal sepsis, given its invasive nature, tissue manipulation, and increased exposure to infection [7,9]. Furthermore, the underlying indications for cesarean section, particularly in emergency settings, may further predispose mothers to infection [14].

The high proportion of mothers who were readmitted after delivering in the hospital may be attributed to the large patient-to-midwife ratios, estimated at approximately 60:1 at night and 50:1 during the day. Such staffing constraints pose significant barriers to the provision of inhospital care, including timely administration of antibiotics and comprehensive patient education, particularly on self-care practices aimed at preventing puerperal sepsis both in the hospital and after discharge. [15]. Limited time for individualized counseling may compromise mothers’ understanding of wound care, hygiene practices, and early recognition of danger signs after discharge [16].

Additionally, limited home support and early resumption of physically demanding activities may impair wound healing. These factors, combined with suboptimal discharge counselling and poor adherence to prescribed antibiotic regimens, likely increase the risk of postdischarge infections. Poor adherence to treatment has also been identified in other studies as a key barrier to effective postpartum health promotion [13].

A study examining maternal age and risk of readmission reported significantly higher postpartum readmission rates among women aged ≥35 years compared to younger mothers. In contrast, our study found that mothers aged 15-19 years had a higher likelihood of readmission compared to those aged ≥35 years. Although maternal age was not identified as a significant factor in other studies [9,12], one study reported low levels of knowledge and poor utilization of postnatal care services among mothers aged 15-25 years [17], which may partly explain the increased risk of readmission in this group. Furthermore, the World Health Organization [16] reports that younger mothers have a higher risk of puerperal sepsis compared to older mothers.

In this study, mothers with lower parity were less likely to be readmitted for puerperal sepsis after discharge. This contrasts with findings from another study, which reported that low-parity mothers are at increased risk of prolonged labor and, consequently, puerperal sepsis [18]. The discrepancy may be explained by greater postnatal attention and support provided to first-time or low-parity mothers. Additionally, factors such as socioeconomic status, social support, and education may influence the risk of puerperal sepsis, although these variables were not fully assessed in our study due to incomplete documentation. Notably, despite the observed protective association, the absolute number of readmissions was higher among primiparous mothers compared to grand multiparous mothers.

Length of hospital stay is an important indicator of disease burden and the level of care required. In this study, mothers who developed puerperal sepsis after discharge had longer hospital stays compared to those who developed sepsis during initial admission. This may be due to delays in seeking care after symptom onset, allowing the infection to progress prior to readmission. Consequently, more advanced disease at presentation may lead to delayed wound healing and increased need for intensive management.

Additionally, community-acquired infections, potentially resulting from poor hygiene practices such as inadequate handwashing, suboptimal personal hygiene, and poor bladder and bowel care, may involve more virulent or antibiotic-resistant organisms compared to some hospital-acquired infections. This can necessitate prolonged antibiotic therapy and extended wound care [19].

Furthermore, we observed a similarly high rate of surgical intervention in both groups. However, a greater number of mothers in the readmitted group underwent hysterectomy compared to those who developed sepsis while still hospitalized. This may reflect delayed diagnosis and more advanced disease at the time of readmission, necessitating interventions such as wound debridement or other surgical management. In contrast, inhospital cases may benefit from earlier detection, prompt initiation of antibiotics, and timely wound care, potentially preventing progression to severe complications requiring major procedures such as hysterectomy.

Our study also found that mortality was higher among mothers who developed puerperal sepsis while hospitalized. This finding is consistent with results from a meta-analysis [20] which examined hospital length of stay and mortality among patients with hospital- and community-acquired sepsis. However, that study focused on critically ill adults, with most included studies conducted in high-income countries, which may explain differences in findings, particularly regarding length of stay.

The higher mortality observed among mothers with inhospital sepsis in our study may be attributed to the presence of significant predisposing factors or comorbidities, which increase vulnerability to severe infection and its complications. These underlying conditions may exacerbate disease progression. In contrast, mothers who develop sepsis after discharge often present with more localized infections, such as wound infections, which, although serious, may be more amenable to treatment, including surgical management when necessary.

Limitations

We relied on patient files and manually extracted data for analysis. Due to gaps in documentation, key demographic variables such as marital status, occupation, employment, and income could not be included in the study. However, we were able to obtain essential demographic information-such as age, parity, and address-which have been identified as important factors in other studies.

A prospective cohort design would have been more appropriate for accurately determining when mothers acquired puerperal sepsis and for minimizing the chances of selection bias due to the availability of controls and direct access to the mothers for comprehensive assessment. However, due to the relatively low number of cases recorded per year, achieving an adequate sample size would have required a prolonged study period, which was not feasible for this study. In this study, we relied on diagnoses made by qualified obstetricians, who were able to justify the diagnosis and obtain relevant clinical histories to describe the course of puerperal sepsis among the mothers.

## Conclusions

Despite the observed reduction in admissions due to puerperal sepsis, the predominance of postdischarge readmissions, particularly among younger mothers and the increased number among those who delivered by cesarean section, remains a significant challenge to achieving Sustainable Development Goal 3. Targeted interventions aimed at improving education on the prevention of puerperal sepsis among young mothers and those who have undergone cesarean section are therefore essential.
